# Snapshot spectral imaging with parallel metasystems

**DOI:** 10.1126/sciadv.abc7646

**Published:** 2020-09-18

**Authors:** Andrew McClung, Sarath Samudrala, Mahsa Torfeh, Mahdad Mansouree, Amir Arbabi

**Affiliations:** Department of Electrical and Computer Engineering, University of Massachusetts Amherst, 151 Holdsworth Way, Amherst, MA 01003, USA.

## Abstract

Spectral imagers divide scenes into quantitative and narrowband spectral channels. They have become important metrological tools in many areas of science, especially remote sensing. Here, we propose and experimentally demonstrate a snapshot spectral imager using a parallel optical processing paradigm based on arrays of metasystems. Our multi-aperture spectral imager weighs less than 20 mg and simultaneously acquires 20 image channels across the 795- to 980-nm spectral region. Each channel is formed by a metasurface-tuned filter and a metalens doublet. The doublets incorporate absorptive field stops, reducing cross-talk between image channels. We demonstrate our instrument’s capabilities with both still images and video. Narrowband filtering, necessary for the device’s operation, also mitigates chromatic aberration, a common problem in metasurface imagers. Similar instruments operating at visible wavelengths hold promise as compact, aberration-free color cameras. Parallel optical processing using metasystem arrays enables novel, compact instruments for scientific studies and consumer electronics.

## Teaser

An array of cascaded metasurfaces implements a compact and lightweight spectral imager that acquires real-time videos.

## INTRODUCTION

Spectral imagers produce images in which each pixel bears quantitative spectral information. Originally developed for astronomy (*1*), spectral imagers now find applications in Earth observation, biomedicine, art conservation, agriculture, and many other scientific and industrial contexts (*2*). Datasets produced by these imagers, called datacubes, map the irradiance of a scene *I*(*x*, *y*, λ) along three axes: two spatial (*x*, *y*) and one spectral (λ). Datacubes are similar to ordinary red-green-blue (RGB) color images, in which each pixel is assigned a red, green, and blue value, but the term typically designates datasets for which color channels are quantitative rather than perceptual, spectrally narrow, and greater in number (*N*_λ_ ≫ 3).

Spectral imaging technologies can be separated into two categories: scanning imagers and snapshot imagers. Scanning spectral imagers acquire several sequential one-dimensional (1D) or 2D measurements to construct the 3D spectral datacube. The scanned dimensions can be either spatial, as with whisk- and push-broom imagers (*3*, *4*), or spectral, as with tunable filter systems (*5*) or imaging Fourier transform spectrometers (*6*). Acquisition of a complete dataset can be slow, particularly in systems with mechanically scanning elements. These long scan periods produce undesirable artifacts in scenes with comparatively quick dynamics (*2*).

By contrast, snapshot spectral imagers acquire a complete dataset in one detector integration period by multiplexing spatiospectral data over a 1D or 2D sensor. These systems require neither internally moving components nor relative motion of imager and object. Snapshot imagers may use dispersive elements (*1*), spectral filters (*7*), or interferometry (*8*) to obtain spectral information. Reference (*2*) surveys a wide range of snapshot imaging architectures.

One conceptually simple snapshot architecture is that of the multi-aperture filtered camera (MAFC) (*7*), which acquires a parallel array of image channels, each with a different spectral filter. In contrast with instruments that use tomographic (*9*) or compressed sensing (*10*) techniques, MAFCs are able to recover the datacube without substantial computation, an advantage in applications that require real-time information or have a low power budget. Moreover, spectral channels in systems based on filters (instead of dispersive elements) need not be adjacent, allowing an instrument to be tailored to application-specific spectral signatures. Multi-aperture instruments have been used for over a century to overcome technical challenges, including high framerate acquisition (*11*), wavefront sensing (*12*), and 3D photography (*13*). More recently, the use of microcamera arrays in multiscale cameras has enabled diffraction-limited gigapixel images (*14*). Data in multi-aperture systems are segmented into channels that can be processed independently, affording advantages analogous to those of parallel computing (*15*).

Metasurface optical components are substantially lighter and more compact than traditional counterparts and can be manufactured using standard nanofabrication tools and techniques, making them an attractive choice for applications with constrained size, weight, or cost. By exerting subwavelength control over phase, polarization, and amplitude of light, metasurfaces can mimic elements like lenses, wave plates, and diffraction gratings (*16*), or implement more exotic optical transformations (*17*–*19*). Improvements in metasurface efficiency have enabled systems of cascaded metasurfaces, or metasystems, that perform sophisticated functions (*20*, *21*). Metasystems could be arrayed compactly and at low marginal cost to reap the benefits of parallelism enjoyed by conventional multi-aperture systems. Here, we refer to these arrays as parallel metasystems.

Although certain optical systems are amenable to miniaturization with metasurfaces, they are not drop-in replacements for refractive elements. An important consideration when designing metasystems is the strong chromatic dispersion of metasurface elements. While chromatic dispersion can be a feature in certain instruments, in imaging systems, it leads to chromatic aberrations, hindering broadband operation. This problem is well known, prompting many proposed solutions (*22*–*24*), but none that scale well to large physical or numerical apertures (NAs).

A metasystem implementing a scanning spectral imager was recently demonstrated (*25*). The instrument, working in the 750- to 850-nm range, operates similarly to a push-broom imager: It images a line object along one axis and disperses it along another, producing one *x*λ plane of the datacube. A complete dataset is acquired by translating the object or imager along the *y* axis. The imager was optimized for spatial and angular resolution, and as a result produces distorted images, requiring characterization and postprocessing to reconstruct the datacubes.

**Fig. 1:**
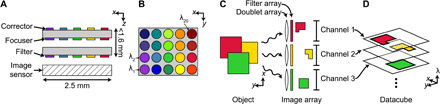
Schematic of proposed MSSI and principle of operation. (**A**) Side view and (**B**) top view. A 4 × 5 array of doublet lenses comprising two metasurfaces is aligned to a matched array of metasurface bandpass filters resting on an image sensor. (**C**) The doublet lens array images an object composed of mixed spectral content. Filters discriminate light traversing the doublet lenses into spectral channels. (**D**) Individual channels are registered to form a 3D spectral datacube.

In this work, we propose and experimentally demonstrate a compact metasurface snapshot spectral imager (MSSI) formed by three cascaded metasurface arrays, the parallel metasystem analog of the MAFC. Figure 1A shows a schematic of our proposed system: The first two metasurface arrays lie on opposite sides of a glass substrate, forming a doublet array in which each pair of metasurfaces implements a lens corrected for monochromatic aberrations. A metasurface filter array (*26*) lies on another glass substrate, comprising bandpass filters with transmission windows matched to the corresponding doublets. Images form 100 μm beyond the second substrate and are captured by an image sensor. The imager’s 20 spectral channels are arranged spatially in wavelength order, as shown in Fig. 1B; each has ca. 7-nm bandwidth and lies in the 795- to 980-nm range. The volume of the entire system excluding the image sensor is less than 8 mm^3^.

Figure 1C illustrates how this system divides a scene into spectral channels: Each doublet forms a polychromatic image, but only components in the passband of the associated filter reach the image plane, resulting in image channels with narrow spectral content. The spectral datacube is assembled by registering and stacking these channels (Fig. 1D). The bandpass filter array, necessary to distinguish spectral channels, also serves a second purpose: Each doublet is corrected at a specific wavelength, far from which image quality degrades due to chromatic aberrations. By suppressing transmission far from the design wavelength, the filters improve image resolution and contrast. In the following, we describe the design and implementation of our MSSI’s constituent elements, including a method to incorporate absorptive field stops. To demonstrate our system, we present several spectral still images and a spectral video.

## RESULTS

**Fig. 2:**
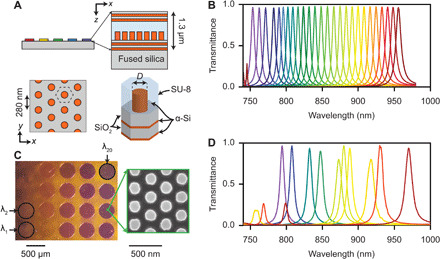
Metasurface bandpass filter array. (**A**) Schematics. Each bandpass filter consists of two DBRs that form a Fabry-Pérot cavity. A layer of SU-8 polymer separates the DBRs, and a uniform array of α-Si nanoposts rests on the lower DBR. Nanopost diameter determines each filter’s passband. (**B**) Simulated transmission passbands for filters with nanopost diameters ranging from 0 to 230 nm. (**C**) Optical micrograph of the completed filter array and electron micrograph of the nanopost array in the filter’s interior (inset). (**D**) Measured transmission passbands for a subset of the filter array.

Our metasurface filters comprise a uniform array of meta-atoms sandwiched between distributed Bragg reflectors (DBRs), forming the modified Fabry-Pérot geometry shown in Fig. 2A. Each DBR consists of two pairs of amorphous silicon (α-Si) and silicon dioxide (SiO_2_) layers. An array of α-Si nanoposts forming a hexagonal lattice rests on the lower DBR layer and is encapsulated above by an SU-8 polymer. The filter array rests on a fused silica substrate. Refractive indices for all filter materials are shown in fig. S1.

Filters with different passbands are obtained by varying nanopost diameter *D*. Figure 2B shows simulated passbands for nanoposts with *D* varying between 0 and 230 nm, exhibiting transmission peaks >95% and full widths at half maximum (FWHMs) between 5 and 8 nm. A photograph of the filter array and an electron micrograph of a portion of one of the filters is shown in Fig. 2C. Details about the filter design and fabrication can be found in Materials and Methods.

Measured transmission spectra for a subset of the filters are shown in Fig. 2D; the complete set of transmission spectra are shown in fig. S2. Fabricated filters exhibit ca. 30-nm redshift from the design and transmission peaks between 77 and 98%. This redshift, the irregular spectral spacing, and the reduced transmission of some filters are attributable to deviations of the thickness of the SU-8 polymer layer and of the nanopost dimensions from their design values (see discussion in Materials and Methods). The free spectral range of each filter is ca. 190 nm; both simulated and measured spectra show the next longitudinal mode for the filters with the longest wavelength passbands.

**Fig. 3:**
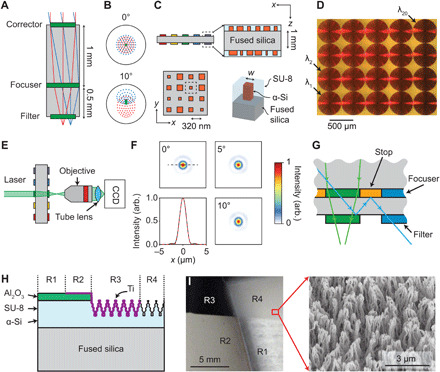
Metasurface doublet array. (**A**) Ray diagrams showing fields incident at 0° and 10°. (**B**) Example spot diagram for fields incident at 0° and 10° for one of the doublets with a design wavelength of λ_0_ = 872.8 nm. Green dots indicate λ_0_, and red and blue dots nearby wavelengths inside the filter passband (λ_0_ ± 4.7 nm). The black circle marks the first zero of the Airy pattern. (**C**) Schematic of doublet array. Both corrector and focuser metasurfaces are implemented by nonuniform, rectangular arrays (320-nm lattice constant) of square, 485-nm-tall α-Si nanoposts encapsulated in SU-8 polymer. (**D**) Optical micrograph showing a portion of the focuser array. (**E**) Measurement setup and (**F**) measured focal spots for light at 0°, 5°, and 10° incidence. Focal spots share an intensity scale. Line graph shows cross section at 0^∘^ (black, solid) with Airy pattern (red, dashed) for comparison. (**G**) Illustration depicting the problem of reflective field stops. An errant ray (blue) rejected by one filter (green) bounces off a reflective field stop (gold) into a neighboring channel, contributing noise. (**H**) Schematic of absorptive apertures showing Al_2_O_3_-protected SU-8 polymer without Ti deposition (R1), Al_2_O_3_-protected SU-8 polymer with Ti deposition (R2), etched SU-8 polymer with Ti deposition (R3), and etched SU-8 polymer without Ti deposition (R4). (**I**) Photo of test sample showing regions labeled in (H) and electron micrograph of etched SU-8 polymer. R1 is transparent, showing the cloth behind the substrate; R2 is opaque and reflective; R3 is opaque and absorptive; and R4 is diffuse. In the electron micrograph, a thin layer of gold was sputtered on the sample to mitigate charging effects.

After characterizing the filter array, we designed a matching doublet lens for each filter. Figure 3A shows a ray optics simulation for one of the lens-filter pairs. The ray tracing model we used treats each element in the doublet as a radially symmetric phase plate. The first element acts as a spherical aberration corrector, while the second performs most of the focusing, as described in (*20*). Here, we refer to these metasurfaces as corrector and focuser. Each doublet is designed to form a diffraction-limited image for an 80° field of view (FOV). The FOV of the fabricated device was limited by a technical constraint: The maximum focuser diameter that could be written in a reasonable time with our electron beam lithography system was 500 μm, restricting the FOV to 20°. The design allows a 4× larger FOV and 2× larger stop aperture diameter, which would substantially increase the system’s space-bandwidth product (*27*). The lens doublet is telecentric in the image space, so the cone of rays focused by the doublet has the same angle of incidence and angular subtense at all points on the filter. Consequently, the filter’s center wavelength is the same across the FOV. Each channel in the system produces images with approximately 58,000 effective pixels (see Materials and Methods).

Figure 3B shows spot diagrams for one of these designs with fields incident at 0° and 10°; marker colors indicate different wavelengths inside the filter passband. All rays fall within the central maximum of the Airy disk (1.8-μm radius) at each incidence angle and wavelength, indicating nearly diffraction-limited performance. The lens doublet’s aperture stop, coplanar with the corrector, has a diameter of 400 μm. This aperture stop and the doublet’s focal length of 678 μm result in an NA of 0.29.

We then designed metasurfaces to realize the corrector and focuser phase plates. Both corrector and focuser metasurfaces consist of 485-nm-tall α-Si nanoposts with square cross section, arranged in a rectangular lattice with 320-nm lattice constant (Fig. 3C). Nanoposts are encapsulated in an SU-8 polymer and rest on a fused silica substrate. Varying the width of the nanoposts in this lattice locally changes the transmitted phase. Detailed descriptions of the doublet array design and fabrication are found in Materials and Methods. Figure 3D shows a micrograph of the corrector array taken before fabricating stops, described below.

Figure 3F shows focal spots for one of the doublet lenses at 0°, 5°, and 10° incidence acquired using the setup shown schematically in Fig. 3E. The line cut of the focal spot at 0° exhibits good agreement with an overlaid Airy pattern, and spots at 5° and 10° incidence show similar peak intensity and diameter, indicating that the lens is well corrected. The lens focuses 76% of the transmitted power (see Materials and Methods).

In our MSSI, we have implemented both aperture and field stops. Stops are openings, typically circular, in an opaque medium intended to prevent undesired rays from reaching an image (*28*). In ordinary, single-aperture cameras, stops are used to reduce aberrations or to modify depth of field. For the design shown in Fig. 3A, the location of the aperture stop determines the coma of the lens, an important factor for image quality. The aperture stops in our system are designed to be coplanar with the corrector metasurfaces. We formed the aperture stops by patterning a 50-nm-thick layer of gold around the corrector metasurfaces via a standard photolithography and lift-off process (see Materials and Methods for details).

Field stops limit the range of incident angles accepted by the system, thus determining the FOV. In our system, the field stops reside on the opposite side of the doublet substrate from the aperture stops, coplanar with the focuser metasurfaces. We could have fabricated the field stops identically to the aperture stops, but placing reflective gold stops adjacent to reflective bandpass filters could potentially introduce stray light into our system (see Fig. 3G). To avoid this, we instead devised a method to create absorptive field stops for the focuser array. Random, high–aspect ratio absorptive nanotextures can be closely impedance matched with free space and have been shown to have very low reflectance (*29*). For our device, we created a process-compatible absorptive layer by etching an SU-8 polymer and coating it with titanium. Antimony, part of the photoinitiator in the SU-8 polymer, produces micromasking (*30*), creating a high–aspect ratio nanotexture when etched in oxygen plasma.

To fabricate the field stops, we first spun on an additional layer of SU-8 polymer on top of the focuser array. We then etched the sample in oxygen plasma, producing a nanotexture that is diffuse but not absorptive. To enhance absorption, we deposited a 40-nm-thick layer of titanium on top of the nanotextured SU-8 polymer. Throughout the process, we protected the metasurfaces to avoid forming an absorptive layer occluding them. Figure 3 (H and I) shows a schematic, photograph, and electron micrograph of textures present on the substrate surface. We measured an absorptance greater than 98% for this material (see Materials and Methods). As a final step, we encapsulated the absorptive layer in the SU-8 polymer to increase durability.

**Fig. 4:**
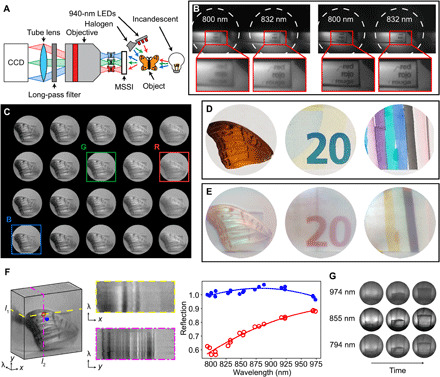
Spectral images and video. (**A**) Schematic of measurement setup. (**B**) Images with filter array retracted (left) and inserted (right). (**C**) Normalized image array. Colored outlines indicate RGB channel assignment for false-color images in (E). (**D**) Visible and (**E**) false-color near-infrared (NIR) images of a butterfly wing (left), Euro banknote (center), and marker pen lines (right). (**F**) Datacube (left) constructed from image array in (C), horizontal and vertical cross sections of datacube (center), and line cuts (right) for corresponding points of interest indicated in datacube, showing spatially variant spectral response. (**G**) Still images captured from spectral video of pouring water. Each row shows a different channel and each column a different time in 1-s increments. Photo credit: Andrew McClung, University of Massachusetts Amherst.

We characterized the MSSI using the setup depicted in Fig. 4A. Instead of placing an image sensor in the MSSI’s image plane, as depicted in Fig. 1A, an objective and a tube lens are used to relay images to a charge-coupled device (CCD) (Fig. 4A). The objective is mounted on a translation stage, allowing us to image different focal planes. To demonstrate that the filter array improves image quality, we compared images with the filter array removed and inserted (Fig. 4B). In both images, we positioned the objective to obtain the best focus in the rightmost spectral channel (832 nm). Without filters in place, the two channels’ planes of best focus do not coincide, as evidenced by the increased blur in the left channel. In the filtered images, by suppressing light far from the doublets’ design wavelengths, we achieve good focus and improved contrast in both channels simultaneously. Figure S3 shows the same comparison with a greater number of channels.

Figure 4C shows all 20 channels of the image array. The center wavelength of each channel increases from bottom to top and left to right; fig. S4 shows the same data with labeled channels. Channels in this image are brightness normalized to correct for uneven illumination and spectral variance of the image sensor’s sensitivity (see Materials and Methods for details). A false-color image can be used to simultaneously visualize the data in three different channels. Figure 4 (D and E) shows visible-wavelength and corresponding false-color near-infrared (NIR) images of various objects. The false-color images are created by assigning the channels indicated in Fig. 4C to red, green, and blue channels (*R* = 974 nm, *G* = 855 nm, and *B* = 794 nm). These images reveal the objects’ chromatic responses in the NIR: The butterfly wing shows uneven reflection; a Euro banknote reveals NIR security features (increased reflection of the numerals at longer wavelengths); and different inks show unexpected absorption in the NIR.

RGB channel mapping is a convenient way to visualize and reason about the NIR behavior of a scene but does not incorporate all information in a spectral image. In Fig. 4F, we show a datacube constructed from the dataset in Fig. 4C, with spatial (*x*, *y*) and spectral (λ) data arranged along orthogonal axes. Slices *l*_1_ (yellow, dashed) and *l*_2_ (magenta, dot dashed) show *x*λ and *y*λ planes, revealing brightness variation across spectral channels. Quantitative point spectra along the λ axis are shown for two points of interest (POIs): The first POI (blue circle, filled) shows a relatively even reflection across the NIR, while the second POI (red circle, open) shows a feature that reflects more at longer wavelengths. Details about datacube assembly are described in Materials and Methods.

As we have already mentioned, one key advantage of snapshot imagers is the simultaneous capture of all spectral data. This feature enabled us to acquire a spectral video, included in movie S1. A few frames from this video, which shows a clear plastic cup being filled with water, are shown in Fig. 4G. Rows correspond to three spectral channels, while columns show frames at 1-s intervals. Increased absorption is evident at longer wavelengths: The water appears dark in the 974-nm channel but transparent in the others. This absorption feature is consistent with the NIR absorption spectrum of water (*31*).

## DISCUSSION

The form factor of the demonstrated spectral imager allows for its potential integration into mobile consumer electronics, such as point-and-shoot cameras, smartphones, or wearable “internet of things” devices. The low weight and small volume of MSSIs also make them attractive for air- or spaceborne instruments carried by unmanned aerial vehicles or CubeSats. Although our MSSI operates in the NIR, analogous visible-wavelength systems could be constructed by using titanium dioxide (*32*) or silicon nitride (*33*) meta-atoms and systems for the mid-infrared by using sapphire substrates (*34*). By placing complementary systems side by side, a multiband imager covering a wide spectral range could be realized.

The MSSI demonstrated in this work has similar spatial resolution in each spectral channel and is designed to have uniformly distributed spectral channels. However, uniform sampling of the datacube may not be desired in all applications: A scene may have greater spatiospectral detail in certain bands and less in others. Spectral sampling could be modified by changing the distribution of filter passbands, and resolution could be selectively enhanced or diminished by increasing or decreasing the size of a channel’s stop aperture.

The MAFC approach described here could also be used to solve the problem of ordinary color imaging with metasurfaces: Mosaic filters (*35*) commonly found in the image plane of color imaging systems do not work for ordinary, single-aperture metasurface imagers because of their narrow operating bandwidth. Spectral images acquired by a visible-wavelength MSSI could be projected onto perceptual color spaces (*36*) to produce RGB or wider gamut images, enabling planar color cameras.

The system presented in this work exemplifies the broader concept of parallel metasystems, multi-aperture arrays of cascaded metasurfaces that work together to process an optical signal. The parallel metasystem architecture is an alternative to single-aperture multifunctional metasurfaces (*37*–*39*) that promises more favorable scaling to a large number of functions. Beyond imaging and spectral filtering, parallel metasystems easily could be designed to measure an optical signal’s fundamental properties, including polarization, phase and degree of coherence, or, using spatial filters (*40*), to perform more sophisticated signal processing, leading to compact implementations of canonical multi-aperture systems and providing a framework for altogether new parallel optical processors.

## MATERIALS AND METHODS

### Simulation and design

The filter array is composed of two DBR mirrors and an SU-8 spacer layer in between (Fig. 2A). A 190-nm-tall layer of α-Si nanoposts was embedded inside and at the bottom of the SU-8 layer and functions as a phase shifter changing the cavity’s resonance wavelength. Each DBR mirror is composed of two pairs of quarter-wave-thick α-Si/SiO_2_ layers. The nanoposts are arranged on a hexagonal lattice, with a lattice constant of 280 nm (Fig. 2A). The entire filter structure is periodic and was simulated using the RCWA (rigorous coupled-wave analysis) technique (*41*). Wavelength-dependent refractive indices for α-Si, SiO_2_, and SU-8 (fig. S1) obtained via ellipsometry (Woollam RC2 Spectroscopic Ellipsometer) were used in these simulations. The cavity length and the height of nanoposts were adjusted such that the resonant transmission peak of the filter spanned the 750- to 950-nm range (Fig. 2A).

Like all interference filters (*42*), the passbands of these filters shift as the angle of incident light deviates from normal. Simulated transmission spectra for one of the filters with light incident at 0° and 15° are shown in fig. S2. The simulated response at 15° incidence exhibits blueshift of around one FWHM (ca. 7 nm) and a reduction in peak transmission from 98 to 92%.

The design of the metalens doublets proceeded as in (*20*). Each doublet was designed at the measured center wavelength of its corresponding filter. To design the doublets, the phase profiles for corrector and focuser were obtained by minimizing the focal spot size for different incident angles using commercial optical design software (Zemax OpticStudio). The effective pixel count per channel, given by the area of the image, π*D*^2^/4 (*D* is diameter of the bandpass filter), divided by the Nyquist sampling period, λ/(2 NA), is approximately 58,000 at λ = 850 nm. To implement the designed phase profiles of the doublets using α-Si nanoposts, we used an RCWA solver (*41*) to simulate the complex transmission amplitudes *t*(*w*) of nanoposts with different widths *w* at the design wavelength of each doublet. From these simulations, we generated design curves *w*(ϕ) relating the optimal nanopost width for a desired transmitted phase ϕ. Here, the “optimal” width is the one that maximizes Re{*t*(*w*)*e*^jϕ^}. A representative design curve for metasurfaces at 860 nm is shown in fig. S5.

### Device fabrication

Filter fabrication began by depositing the lower DBR and metasurface device layers. Without breaking vacuum, we deposited the DBR—four alternating layers of plasma-enhanced chemical vapor deposition (PECVD) α-Si (51 nm) and SiO_2_ (135 nm)—and the α-Si device layer (190 nm) on top of a 500-μm fused silica substrate. This and all PECVD processes described below were run at 300°C. To pattern the metasurface layer, we first spun a positive-tone electron beam resist (Zeon ZEP 520A-7) and a conductive polymer (Allresist AR-PC 5090) on top of the substrate. We exposed the filter pattern in a 125-keV electron beam lithography system (Elionix ELS-F125). To form an etch mask, we deposited ca. 50 nm of Al_2_O_3_ on top of the resist in an electron beam evaporator. We lifted off in solvent (MicroChem Remover PG) at 80°C and transferred the pattern to the device layer by reactive ion etching in SF_6_ and C_4_F_8_ gases. The Al_2_O_3_ was removed in a heated solution of ammonium hydroxide and hydrogen peroxide. To encapsulate the nanoposts and planarize the sample, we spun on a layer of SU-8 polymer (MicroChem) to fill the gaps between the nanoposts and to achieve a 435-nm-thick layer on top of the nanoposts. We used ultraviolet (UV) light to cross-link the SU-8 polymer and baked the sample on a hotplate at 200°C, making the layer permanent. Last, we deposited the top DBR, identical to the first, on top of the SU-8 polymer layer.

To fabricate the doublet lens array, we first deposited 485 nm of PECVD α-Si on each side of a 1-mm-thick fused silica substrate. We then defined the metasurfaces using the same process as above, this time, with a 5-μm SU-8 polymer layer to encapsulate. To ensure adequate alignment of focuser and corrector arrays, we created registration marks on the corrector side of the substrate by evaporating gold into a negative photoresist lift-off mask (Futurrex NR9-1000PY). The lift-off mask was aligned to the focuser metasurfaces using backside alignment photolithography (Süss MicroTec MA6). Lift-off was performed in acetone. We used the alignment marks to register the corrector pattern during electron beam lithography and fabricated the array in a process otherwise identical to the focuser array.

We created aperture stops on both sides of the doublet lens array, beginning with the nanotextured absorptive stops on the focuser array. This process began by spinning an additional 5-μm layer of SU-8 polymer on top of the focuser array, exposing it with UV, cross-linking, and curing it. To maintain smooth facets above the lenses, we evaporated a 120-nm Al_2_O_3_ etch mask into a negative photolithography mask (Futurrex NR9-1000PY), lifting off to selectively mask the SU-8 polymer above the metasurfaces. We then etched the sample in oxygen in a reactive ion etcher, producing the nanotexture shown in Fig. 3I. Next, we deposited titanium on the etched SU-8 polymer surface. To mask the lens facets, we first patterned a positive photoresist (EMD AZ3318D) before sputtering 40 nm of titanium on top of the sample. We lifted off the mask in a solvent (MicroChem Remover PG) and encapsulated the absorptive layers in an additional 5-μm cured SU-8 polymer layer. For the corrector array, we created 90-nm gold aperture stops using a photolithography mask (Futurrex NR9-1000PY), evaporation, and lift-off. Five nanometers of chrome was used as an adhesion layer for the gold. After lift-off, an additional 5-μm layer of cured SU-8 polymer was spun on top to protect the gold.

### Device characterization

We measured filter transmission spectra using a broadband white light source (NKT SuperK COMPACT) and optical spectrum analyzer (Ando AQ6317). To normalize these spectra, we measured the transmitted power of 969- and 970-nm filters using a tunable laser (Spectra Diode Labs SDL-TC30) and a calibrated power meter (Newport 1918-C with 918D-SL-OD1 head). A knife edge measurement of the laser beam gave an *e*^−2^ radius of 50 μm. We confirmed that the filter is uniform by measuring the laser transmission at a few points on the filter surface. Optical setups for filter characterization, a spectrum of the white light source, and transmission spectra for all 20 filters are shown in fig. S2. Two fabrication nonidealities can produce the redshift we see with respect to the design: A thicker layer of SU-8 polymer can increase the intracavity length, shifting the passband; a bias in the nanopost width can also produce such a shift. The shifts of the filter passbands relative to their designed values are shown in fig. S2. The spatial correlation of these shifts indicates a nonuniform SU-8 spacer layer thickness or errors in the nanopost dimensions (possibly the result of proximity effects). The reduced transmission of some filters can be attributed to random variation of nanopost shape and width.

The schematic in Fig. 3E shows the basic optical setup for imaging focal spots at different incidence angles and for measuring lens efficiency. Images of focal spots were relayed by a 50× objective (Olympus LMPlanFL N 50×/0.50) and a 200-mm tube lens (Thorlabs ITL200) and captured by a low-noise camera (Photometrics CoolSnap K4). A collimated 850-nm laser was used to illuminate the lens aperture; different incident angles were obtained by moving the source.

To characterize the focusing efficiency, we measured the fraction of power focused by the lens that passes through a 10-μm-diameter pinhole located in the focal plane. We made the pinhole in an aluminum film evaporated on a 500-μm glass substrate. We measured the ratio of light passing through the pinhole to all transmitted power, adjusted for 4% reflection at two glass-air interfaces on the aperture, to be 0.76. Optical powers were measured using a calibrated power meter (ILX Lightwave OMM-6810B with an OMH-6722 power head). Near the lens doublets, we patterned an empty pair of aperture and field stops (i.e., the same structure as the doublets but without nanoposts). We measured the ratio of power passing through the lens doublet to the power passing through the empty aperture and field stops to be 0.56.

The transmittance and reflectance of the absorptive material used to create the aperture stops were measured using a 632.8-nm helium-neon laser (JDS Uniphase 1122) and an integrating sphere (Newport 819-IS-2). A schematic of the measurement setup is shown in fig. S6. We measured direct transmission by placing the sample at a long distance (12 cm) in front of the integrating sphere, diffuse transmission by placing the sample directly in front of the 0° port and unplugging the 180° port, and the sum of specular and diffuse reflection by placing the sample directly behind the 180° port at a shallow angle (ca. 5°). We measured direct transmission, diffuse transmission, and reflection of 0.3, 0.2, and 0.9%, indicating an absorptance of 98.6%.

### Measurement and datacube assembly

The datacube acquisition setup is shown schematically in Fig. 4A. We placed objects at a distance between 5 and 20 cm, allowing them to fill the MSSI’s FOV. Reflective objects shown in Fig. 4 (B, E, and F) were illuminated by a 10-W halogen lamp (Philips 046677417222) and, to boost the intensity at longer wavelengths, a pack of 940-nm light-emitting diodes (LEDs) (SCS IR940B-48). Transmissive objects (Fig. 4G) were illuminated from behind by a 29-W halogen bulb (EcoSmart 52605) and white paper diffuser. The halogen sources specify color temperatures of 2800 and 2790 K, respectively. Measured spectra for all sources are shown in fig. S7. The image relay system consists of a microscope objective, 800-nm long-pass filter (Thorlabs FEL0800), and a tube lens with 150-mm focal length (Thorlabs AC254–150-AB-ML); the camera used in device characterization was also used to acquire images. To enhance detail in Fig. 4B and fig. S3, a 10× objective (Olympus UPlanFL N 10×/0.30) was used; however, the FOV of this objective is smaller than the MSSI image area. For Fig. 4 (C, E, F, and G), a 5× objective (Zeiss Fluar 5×/0.25) was used instead.

The data presented in Fig. 4 (C to G) are brightness normalized. This is necessary because our illumination is spatially nonuniform and also because the quantum efficiency of our image sensor varies substantially across the studied wavelength range (see fig. S8). An unnormalized image is shown in fig. S9. Normalization is achieved by elementwise division of raw datasets by an image of a white object (here a sheet of printer paper).

Because each of the MSSI image channels has a slightly different view of the object, we performed parallax correction before overlaying channels. To do this, we placed a calibration object at the same distance as the objects presented in Fig. 4. We determined coordinates of point features in each channel using an image processing program (*43*) and determined an affine transform between the point features in the 855-nm channel and in each other channel; that is, for each channel *c*, we determine a matrix *t_ij_* that relates the coordinates in channel *c* to the coordinates in the 855-nm channel

$$\left(\begin{array}{c} x_{c}\\ y_{c} \end{array})=(\begin{array}{ccc} t_{11} & t_{12} & t_{13}\\ t_{21} & t_{22} & t_{23} \end{array})(\begin{array}{c} x_{855}\\ y_{855}\\ 1 \end{array}\right)$$ (1)

The transform coefficients *t_ij_* are obtained by minimizing the Euclidean distance between the coordinates obtained by transform and the measured coordinates. Figure S10 shows the parallax calibration image and identifies a subset of the analogous features in three of the channels.

## Supplementary Material

abc7646_Movie_S1.avi

abc7646_SM.pdf

## SUPPLEMENTARY MATERIALS

Supplementary material for this article is available at http://advances.sciencemag.org/cgi/content/full/6/38/eabc7646/DC1
