# Supplementary material for: Snapshot spectral imaging with parallel metasystems
Source: Sci Adv. 2020 Sep 18;6(38):eabc7646. doi: 10.1126/sciadv.abc7646 (PMC7500936; doi:10.1126/sciadv.abc7646)
Supplement: abc7646_SM.pdf [file abc7646_SM.pdf]

Science Advances
AAAS

advances.sciencemag.org/cgi/content/full/6/38/eabc7646/DC1

Supplementary Materials for

Snapshot spectral imaging with parallel metasystems

Andrew McClung, Sarath Samudrala, Mahsa Torfeh, Mahdad Mansouree, Amir Arbabi*

*Corresponding author. Email: arbabi@umass.edu

Published 18 September 2020, *Sci. Adv.* **6**, eabc7646 (2020)
DOI: 10.1126/sciadv.abc7646

The PDF file includes:

Figs. S1 to S10
References

Other Supplementary Material for this manuscript includes the following:

(available at advances.sciencemag.org/cgi/content/full/6/38/eabc7646/DC1)

Movie S1

Supplementary Figures

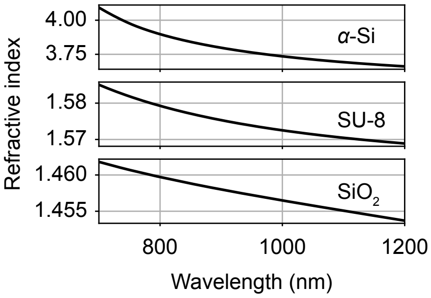

Fig. S1. Refractive indices of filter materials. Data was obtained via ellipsometry (see Methods).

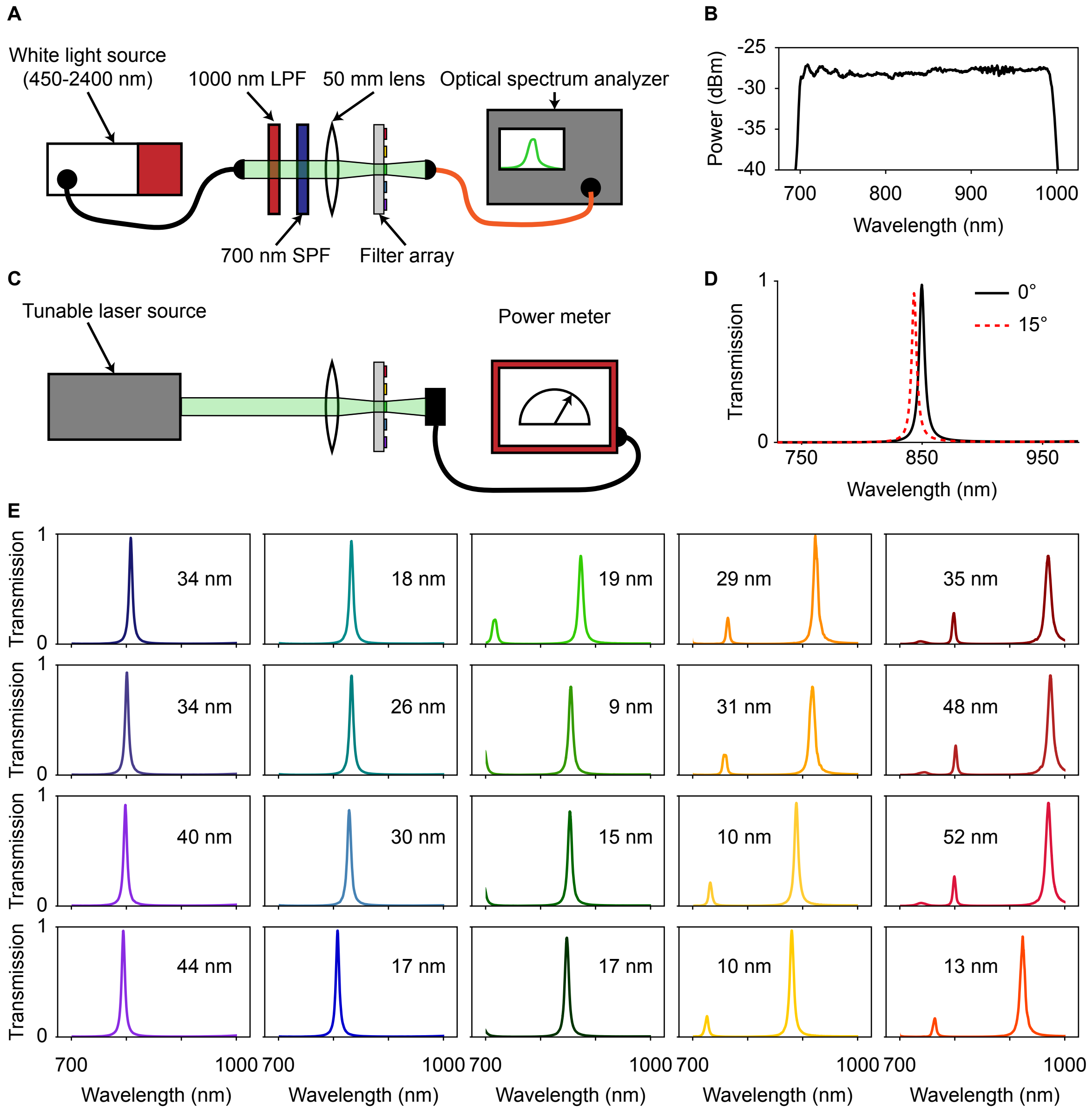

Fig. S2. Filter characterization, angular dependence and transmission spectra. (**A**) Optical characterization of filters using white light source. Long (LPF) and short pass (SPF) filters were used to suppress light outside of the 700–1000 nm band. (**B**) Spectrum of white light source with filters removed. (**C**) A tunable laser source (955–1010 nm) was used to accurately measure the peak transmission of one of the filters and to normalize spectra obtained with the white light source. (**D**) Simulated transmission spectra for a filter designed for 860 nm. The filter is designed for normal incidence (solid, black). At 15° from normal (red, dashed) the passband blueshifts and peak transmission slightly decreases. (**E**) Spectra arranged spatially in channel order, corresponding to data in Fig. 4C and Fig. S4. Redshifts of filter center wavelengths with respect to designed values are indicated.

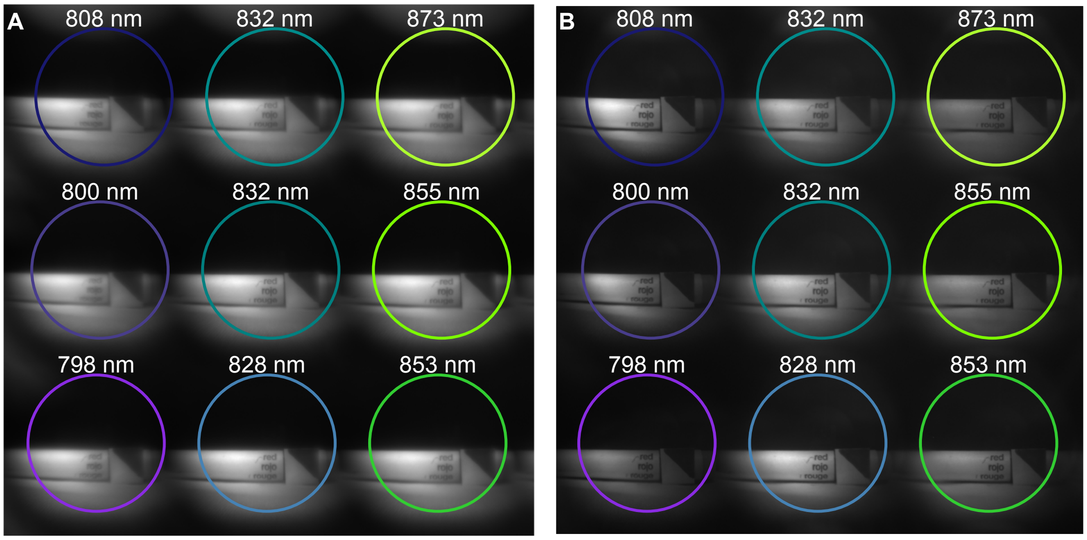

Fig. S3. Suppressing chromatic aberration. Comparison of image channels (**A**) without and (**B**) with filter array in place.

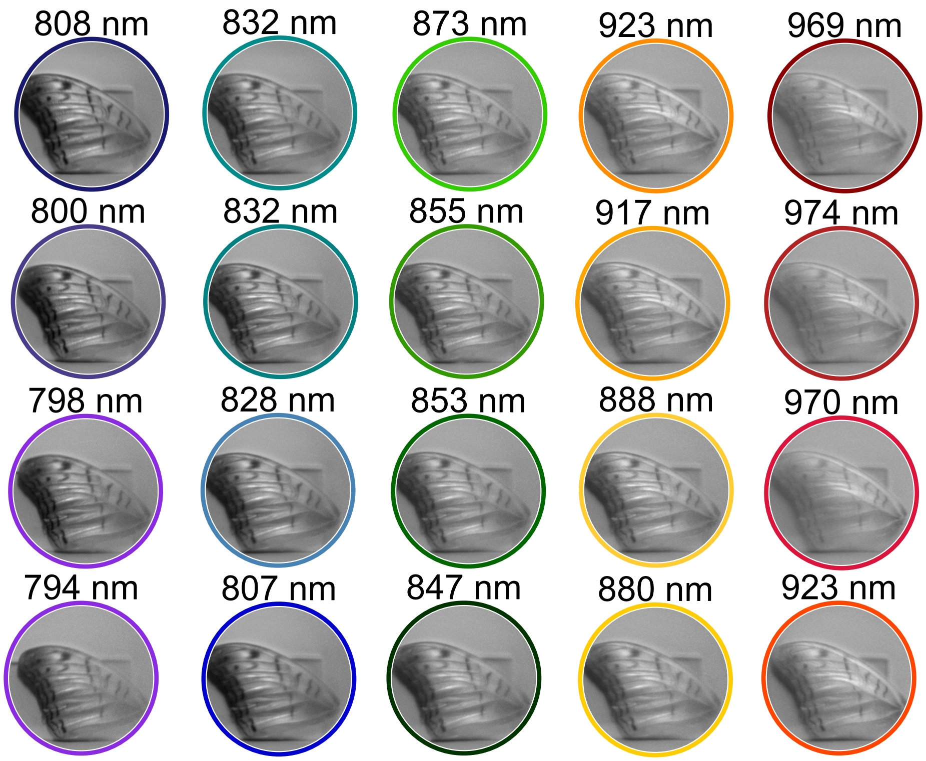

Fig. S4. Channel center wavelengths. MSSI channels labeled by center wavelength. Spatial order corresponds to that of Fig. 4C and Fig. S2.

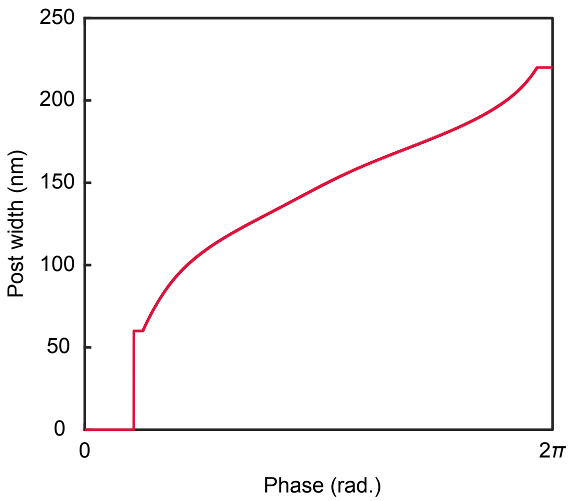

Fig. S5. Metalens design curve. Optimized post width as a function of phase for corrector and focuser metasurfaces comprising lens doublet with design wavelength of 860 nm.

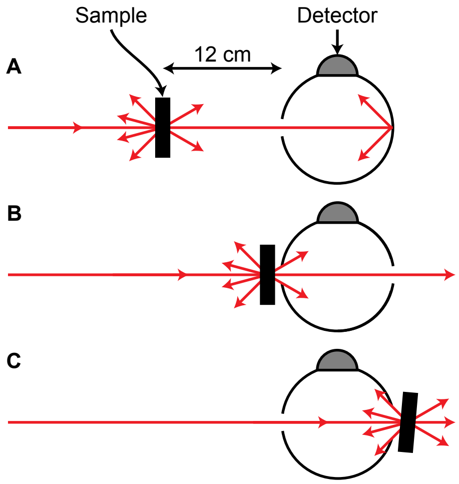

Fig. S6. Transmittance and reflectance measurement. Optical properties of the absorptive layer were characterized using an integrating sphere (see Methods). Schematics show measurement setups for (**A**) direct transmittance, (**B**) diffuse transmittance, and (**C**) reflectance.

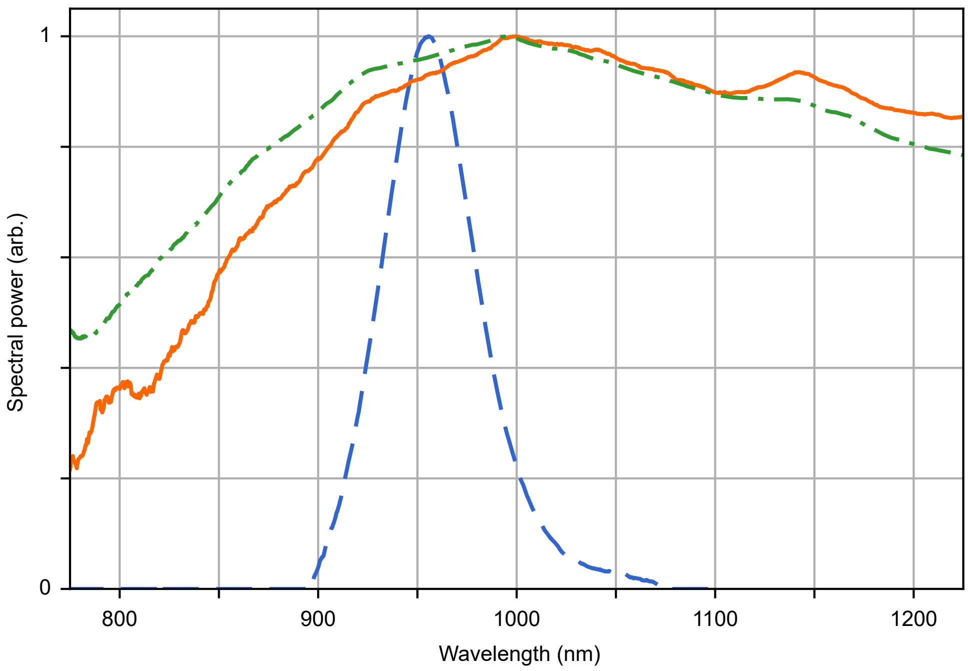

Fig. S7. Source spectra. Spectral power for LED source (blue, dashed), Philips halogen (orange, solid) and EcoSmart halogen (green, dot dashed).

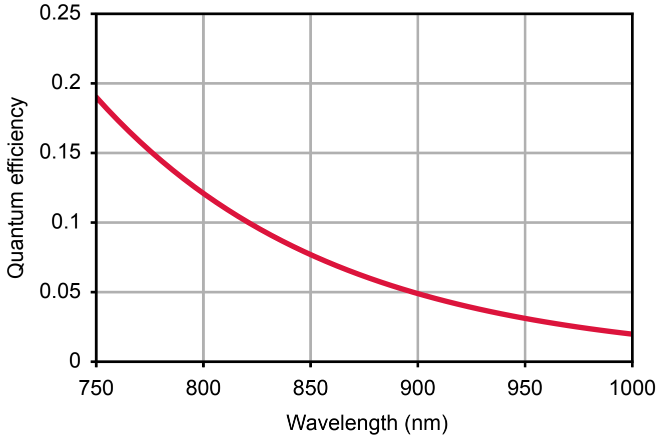

Fig. S8. Quantum efficiency of image sensor. Approximate quantum efficiency of CoolSNAP K4 image sensor. Quantum efficiency changes by a multiplicative factor of ca. 4 across the studied spectral range. See datasheet (44) for full information.

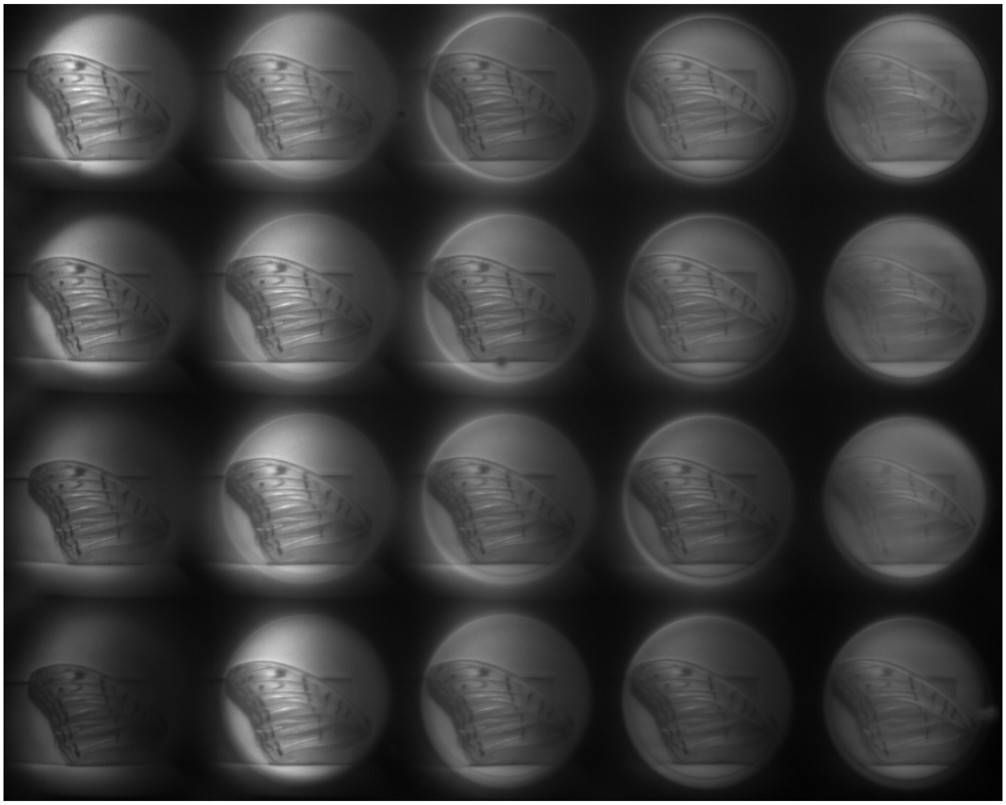

Fig. S9. Unprocessed image. This image contains the data in Fig. 4C before normalizing. Non-uniform illumination is evident; normalization also corrects for decreased quantum efficiency of our image sensor at long wavelengths (see Fig. S8).

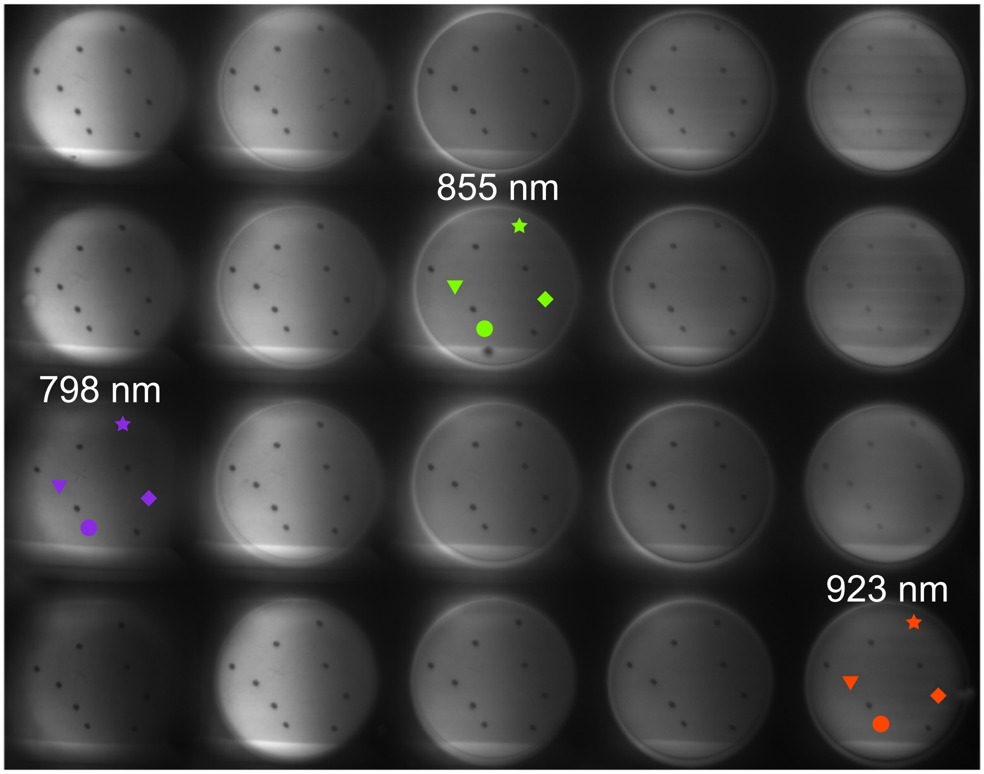

Fig. S10. Affine transform calibration image. We determine an affine transform by identifying analogous features in different spectral channels. In this figure, the star, triangle, circle and diamond symbols in different channels indicate analogous features.

REFERENCES AND NOTES

1. I. S. Bowen, The image-slicer, a device for reducing loss of light at slit of stellar spectrograph. *Astrophys. J.* **88**, 113 (1938).

2. N. Hagen, M. W. Kudenov, Review of snapshot spectral imaging technologies. *Opt. Eng.* **52**, 090901 (2013).

3. P. Mouroulis, R. O. Green, T. G. Chrien, Design of pushbroom imaging spectrometers for optimum recovery of spectroscopic and spatial information. *Appl. Opt.* **39**, 2210–2220 (2000).

4. J. R. Irons, J. L. Dwyer, J. A. Barsi, The next Landsat satellite: The Landsat data continuity mission. *Remote Sens. Environ.* **122**, 11–21 (2012).

5. N. Gat, *Wavelet Applications VII* (International Society for Optics and Photonics, 2000), vol. 4056, pp. 50–64.

6. C. L. Bennett, M. R. Carter, D. J. Fields, J. A. M. Hernandez, *Imaging Spectrometry of the Terrestrial Environment* (International Society for Optics and Photonics, 1993), vol. 1937, pp. 191–200.

7. R. Shogenji, Y. Kitamura, K. Yamada, S. Miyatake, J. Tanida, Multispectral imaging using compact compound optics. *Opt. Express* **12**, 1643–1655 (2004).

8. M. W. Kudenov, M. E. Jungwirth, E. L. Dereniak, G. R. Gerhart, White-light Sagnac interferometer for snapshot multispectral imaging. *Appl. Opt.* **49**, 4067–4076 (2010).

9. M. Descour, E. Dereniak, Computed-tomography imaging spectrometer: Experimental calibration and reconstruction results. *Appl. Opt.* **34**, 4817–4826 (1995).

10. A. Wagadarikar, R. John, R. Willett, D. Brady, Single disperser design for coded aperture snapshot spectral imaging. *Appl. Opt.* **47**, B44–B51 (2008).

11. J. Muybridge, The horse in motion. *Nature* **25**, 605 (1882).

12. J. Hartmann, Objektivuntersuchungen. *Z. Instrumentenkd.* **24**, 1 (1904).

13. G. Lippmann, Épreuves réversibles. photographics intégrales. *C. R. Acad. Sci.* **146**, 446 (1908).

14. D. J. Brady, M. E. Gehm, R. A. Stack, D. L. Marks, D. S. Kittle, D. R. Golish, E. M. Vera, S. D. Feller, Multiscale gigapixel photography. *Nature* **486**, 386–389 (2012).

15. D. J. Brady, W. Pang, H. Li, Z. Ma, Y. Tao, X. Cao, Parallel cameras. *Optica* **5**, 127–137 (2018).

16. D. Lin, P. Fan, E. Hasman, M. L. Brongersma, Dielectric gradient metasurface optical elements. *Science* **345**, 298–302 (2014).

17. D. Wen, F. Yue, G. Li, G. Zheng, K. Chan, S. Chen, M. Chen, K. F. Li, P. W. H. Wong, K. W. Cheah, E. Yue Bun Pun, S. Zhang, X. Chen, Helicity multiplexed broadband metasurface holograms. *Nat. Commun.* **6**, 8241 (2015).

18. S. M. Kamali, E. Arbabi, A. Arbabi, Y. Horie, M. S. Faraji-Dana, A. Faraon, Angle-multiplexed metasurfaces: Encoding independent wavefronts in a single metasurface under different illumination angles. *Phys. Rev. X*, **7**, 041056 (2017).

19. Y. Zhou, I. I. Kravchenko, H. Wang, H. Zheng, G. Gu, J. Valentine, Multifunctional metaoptics based on bilayer metasurfaces. *Light Sci. Appl.* **8**, 80 (2019).

20. A. Arbabi, E. Arbabi, S. M. Kamali, Y. Horie, S. Han, A. Faraon, Miniature optical planar camera based on a wide-angle metasurface doublet corrected for monochromatic aberrations. *Nat. Commun.* **7**, 13682 (2016).

21. M. Faraji-Dana, E. Arbabi, A. Arbabi, S. M. Kamali, H. Kwon, A. Faraon, Compact folded metasurface spectrometer. *Nat. Commun.* **9**, 4196 (2018).

22. E. Arbabi, A. Arbabi, S. M. Kamali, Y. Horie, A. Faraon, Controlling the sign of chromatic dispersion in diffractive optics with dielectric metasurfaces. *Optica* **4**, 625–632 (2017).

23. O. Avayu, E. Almeida, Y. Prior, T. Ellenbogen, Composite functional metasurfaces for multispectral achromatic optics. *Nat. Commun.* **8**, 14992 (2017).

24. M. Khorasaninejad, Z. Shi, A. Y. Zhu, W. T. Chen, V. Sanjeev, A. Zaidi, F. Capasso, Achromatic metalens over 60 nm bandwidth in the visible and metalens with reverse chromatic dispersion. *Nano Lett.* **17**, 1819–1824 (2017).

25. M. Faraji-Dana, E. Arbabi, H. Kwon, S. M. Kamali, A. Arbabi, J. G. Bartholomew, A. Faraon, Hyperspectral imager with folded metasurface optics. *ACS Photonics* **6**, 2161–2167 (2019).

26. Y. Horie, A. Arbabi, E. Arbabi, S. M. Kamali, A. Faraon, Wide bandwidth and high resolution planar filter array based on DBR-metasurface-DBR structures. *Opt. Express* **24**, 11677–11682 (2016).

27. A. W. Lohmann, R. G. Dorsch, D. Mendlovic, Z. Zalevsky, C. Ferreira, Space–bandwidth product of optical signals and systems. *J. Opt. Soc. Am. A* **13**, 470–473 (1996).

28. M. Born, E. Wolf. *Principles of Optics* (Cambridge Univ. Press, Cambridge, ed. 7, 1999).

29. S. Koynov, M. S. Brandt, M. Stutzmann, Black nonreflecting silicon surfaces for solar cells. *Appl. Phys. Lett.* **88**, 203107 (2006).

30. K. H. Rasmussen, S. S. Keller, F. Jensen, A. M. Jorgensen, O. Hansen, SU-8 etching in inductively coupled oxygen plasma. *Microelectron. Eng.* **112**, 35–40 (2013).

31. L. Kou, D. Labrie, P. Chylek, Refractive indices of water and ice in the 0.65- to 2.5-μm spectral range. *Appl. Opt.* **32**, 3531–3540 (1993).

32. R. C. Devlin, M. Khorasaninejad, W. T. Chen, J. Oh, F. Capasso, Broadband high-efficiency dielectric metasurfaces for the visible spectrum. *Proc. Natl. Acad. Sci. U.S.A.* **113**, 10473–10478 (2016).

33. A. Zhan, S. Colburn, R. Trivedi, T. K. Fryett, C. M. Dodson, A. Majumdar, Low-contrast dielectric metasurface optics. *ACS Photonics* **3**, 209–214 (2016).

34. A. Arbabi, R. M. Briggs, Y. Horie, M. Bagheri, A. Faraon, Efficient dielectric metasurface collimating lenses for mid-infrared quantum cascade lasers. *Opt. Express* **23**, 33310–33317 (2015).

35. B. E. Bayer, Color imaging array, US patent 3971065 (1976).

36. A. R. Robertson, The CIE 1976 color-difference formulae. *Color. Res. Appl.* **2**, 7–11 (1977).

37. W. Zhao, B. Liu, H. Jiang, J. Song, Y. Pei, Y. Jiang, Full-color hologram using spatial multiplexing of dielectric metasurface. *Opt. Lett.* **41**, 147–150 (2016).

38. E. Arbabi, A. Arbabi, S. M. Kamali, Y. Horie, A. Faraon, Multiwavelength polarization-insensitive lenses based on dielectric metasurfaces with meta-molecules. *Optica* **3**, 628–633 (2016).

39. J. Ding, S. An, B. Zheng, H. Zhang, Multiwavelength metasurfaces based on single-layer dual-wavelength meta-atoms: Toward complete phase and amplitude modulations at two wavelengths. *Adv. Opt. Mater.* **5**, 1700079 (2017).

40. A. V. Lugt, Signal detection by complex spatial filtering. *IEEE Trans. Inf. Theory* **10**, 139–145 (1964).

41. V. Liu, S. Fan, S^4: A free electromagnetic solver for layered periodic structures. *Comput. Phys. Commun.* **183**, 2233–2244 (2012).

42. P. H. Lissberger, W. L. Wilcock, Properties of all-dielectric interference filters. II. Filters in parallel beams of light incident obliquely and in convergent beams. *J. Opt. Soc. Am.* **49**, 126–130 (1959).

43. J. Schindelin, I. Arganda-Carreras, E. Frise, V. Kaynig, M. Longair, T. Pietzsch, S. Preibisch, C. Rueden, S. Saalfeld, B. Schmid, J.-Y. Tinevez, D. J. White, V. Hartenstein, K. Eliceiri, P. Tomancak, A. Cardona, Fiji: An open-source platform for biological-image analysis. *Nat. Methods* **9**, 676–682 (2012).

44. Photometrics, *CoolSNAP™ K4 Monochrome Datasheet* (2010).
